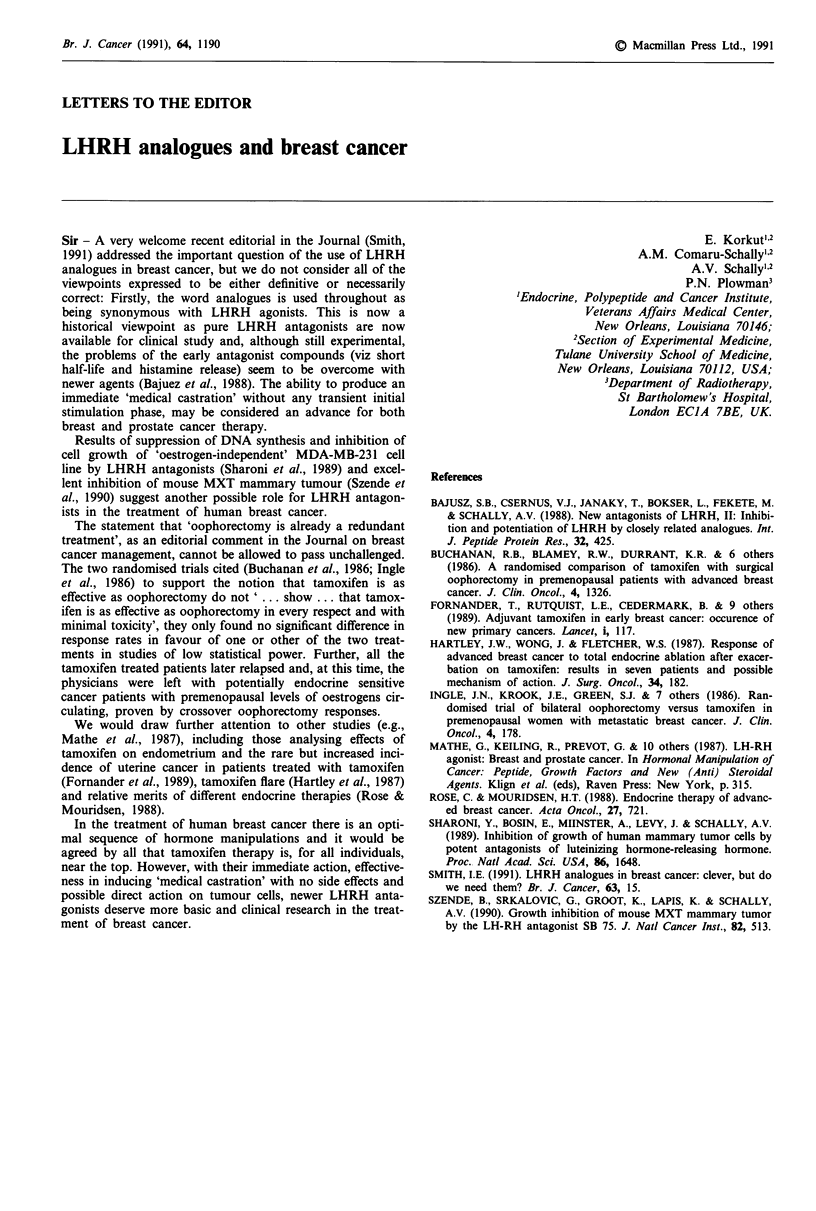# LHRH analogues and breast cancer.

**DOI:** 10.1038/bjc.1991.490

**Published:** 1991-12

**Authors:** E. Korkut, A. M. Comaru-Schally, A. V. Schally, P. N. Plowman


*Br. J. Cancer* (1991), **64**, 1190 © Macmillan Press Ltd., 1991

### LETTERS TO THE EDITOR

### LHRH analogues and breast cancer

**Sir** – A very welcome recent editorial in the Journal (Smith, 1991) addressed the important question of the use of LHRH analogues in breast cancer, but we do not consider all of the viewpoints expressed to be either definitive or necessarily correct: Firstly, the word analogues is used throughout as being synonymous with LHRH agonists. This is now a historical viewpoint as pure LHRH antagonists are now available for clinical study and, although still experimental, the problems of the early antagonist compounds (viz short half-life and histamine release) seem to be overcome with newer agents (Bajuez *et al*., 1988). The ability to produce an immediate 'medical castration' without any transient initial stimulation phase, may be considered an advance for both breast and prostate cancer therapy.

Results of suppression of DNA synthesis and inhibition of cell growth of 'oestrogen-independent' MDA-MB-231 cell line by LHRH antagonists (Sharoni *et al*., 1989) and excellent inhibition of mouse MXT mammary tumour (Szende *et al*., 1990) suggest another possible role for LHRH antagonists in the treatment of human breast cancer.

The statement that 'oophorectomy is already a redundant treatment', as an editorial comment in the Journal on breast cancer management, cannot be allowed to pass unchallenged. The two randomised trials cited (Buchanan *et al*., 1986; Ingle *et al*., 1986) to support the notion that tamoxifen is as effective as oophorectomy do not '... show ... that tamoxifen is as effective as oophorectomy in every respect and with minimal toxicity', they only found no significant difference in response rates in favour of one or other of the two treatments in studies of low statistical power. Further, all the tamoxifen treated patients later relapsed and, at this time, the physicians were left with potentially endocrine sensitive cancer patients with premenopausal levels of oestrogens circulating, proven by crossover oophorectomy responses.

We would draw further attention to other studies (e.g., Mathe *et al*., 1987), including those analysing effects of tamoxifen on endometrium and the rare but increased incidence of uterine cancer in patients treated with tamoxifen (Fornander *et al*., 1989), tamoxifen flare (Hartley *et al*., 1987) and relative merits of different endocrine therapies (Rose & Mouridsen, 1988).

In the treatment of human breast cancer there is an optimal sequence of hormone manipulations and it would be agreed by all that tamoxifen therapy is, for all individuals, near the top. However, with their immediate action, effectiveness in inducing 'medical castration' with no side effects and possible direct action on tumour cells, newer LHRH antagonists deserve more basic and clinical research in the treatment of breast cancer.

E. Korkut[1,2]
A.M. Comaru-Schally[1,2]
A.V. Schally[1,2]
P.N. Plowman[3]
[1]*Endocrine, Polypeptide and Cancer Institute, Veterans Affairs Medical Center, New Orleans, Louisiana 70146;*
[2]*Section of Experimental Medicine, Tulane University School of Medicine, New Orleans, Louisiana 70112, USA;*
[3]*Department of Radiotherapy, St Bartholomew's Hospital, London EC1A 7BE, UK.*

### References

BAJUSZ, S.B., CSERNUS, V.J., JANAKY, T., BOKSER, L., FEKETE, M. & SCHALLY, A.V. (1988). New antagonists of LHRH, II: Inhibition and potentiation of LHRH by closely related analogues. *Int. J. Peptide Protein Res*., **32**, 425.

BUCHANAN, R.B., BLAMEY, R.W., DURRANT, K.R. & 6 others (1986). A randomised comparison of tamoxifen with surgical oophorectomy in premenopausal patients with advanced breast cancer. *J. Clin. Oncol*., **4**, 1326.

FORNANDER, T., RUTQUIST, L.E., CEDERMARK, B. & 9 others (1989). Adjuvant tamoxifen in early breast cancer: occurence of new primary cancers. *Lancet*, **i**, 117.

HARTLEY, J.W., WONG, J. & FLETCHER, W.S. (1987). Response of advanced breast cancer to total endocrine ablation after exacerbation on tamoxifen: results in seven patients and possible mechanism of action. *J. Surg. Oncol*., **34**, 182.

INGLE, J.N., KROOK, J.E., GREEN, S.J. & 7 others (1986). Randomised trial of bilateral oophorectomy versus tamoxifen in premenopausal women with metastatic breast cancer. *J. Clin. Oncol*., **4**, 178.

MATHE, G., KEILING, R., PREVOT, G. & 10 others (1987). LH-RH agonist: Breast and prostate cancer. In *Hormonal Manipulation of Cancer: Peptide, Growth Factors and New (Anti) Steroidal Agents*. Klign *et al*. (eds), Raven Press: New York, p. 315.

ROSE, C. & MOURIDSEN, H.T. (1988). Endocrine therapy of advanced breast cancer. *Acta Oncol*., **27**, 721.

SHARONI, Y., BOSIN, E., MIINSTER, A., LEVY, J. & SCHALLY, A.V. (1989). Inhibition of growth of human mammary tumor cells by potent antagonists of luteinizing hormone-releasing hormone. *Proc. Natl Acad. Sci. USA*, **86**, 1648.

SMITH, I.E. (1991). LHRH analogues in breast cancer: clever, but do we need them? *Br. J. Cancer*, **63**, 15.

SZENDE, B., SRKALOVIC, G., GROOT, K., LAPIS, K. & SCHALLY, A.V. (1990). Growth inhibition of mouse MXT mammary tumor by the LH-RH antagonist SB 75. *J. Natl Cancer Inst*., **82**, 513.